# Aircraft Landing Gear Retraction/Extension System Fault Diagnosis with 1-D Dilated Convolutional Neural Network

**DOI:** 10.3390/s22041367

**Published:** 2022-02-10

**Authors:** Jie Chen, Qingshan Xu, Yingchao Guo, Runfeng Chen

**Affiliations:** 1 School of Civil Aviation, Northwestern Polytechnical University, Xi’an 710072, China; niguanfeixian1994@163.com (Q.X.); guo_ying_chao2020@163.com (Y.G.); 2 China Academy of Space Technology (CAST), Beijing 100081, China; chenrunfeng2018@126.com

**Keywords:** landing gear retraction/extension(R/E) system, 1-D dilated convolutional neural network (1-DDCNN), fault diagnosis, feature integration

## Abstract

The faults of the landing gear retraction/extension(R/E) system can result in the deterioration of an aircraft’s maneuvering conditions; how to identify the faults of the landing gear R/E system has become a key issue for ensuring aircraft take-off and landing safety. In this paper, we aim to solve this problem by proposing the 1-D dilated convolutional neural network (1-DDCNN). Aiming at developing the limited feature information extraction and inaccurate diagnosis of the traditional 1-DCNN with a single feature, the 1-DDCNN selects multiple feature parameters to realize feature integration. The performance of the 1-DDCNN in feature extraction is explored. Importantly, using padding dilated convolution to multiply the receptive field of the convolution kernel, the 1-DDCNN can completely retain the feature information in the original signal. Experimental results demonstrated that the proposed method has high accuracy and robustness, which provides a novel idea for feature extraction and fault diagnosis of the landing gear R/E system.

## 1. Introduction

The landing gear R/E system is the significant subsystem for aircrafts, after long-term running under complex and variable conditions, with heavy loads and strong impact, the key parts in the landing gear R/E system will inevitably generate multifarious faults, which may affect take-off, landing, and flight safety.

Firstly, Hinton proposed a deep learning method in 2006, which set off a new wave of research on artificial intelligence and its applications [1]. In particular, deep learning models have shown significant success in image processing, speech recognition, target detection, information retrieval, natural language processing, and so on [2]. Moreover, as an important network structure, CNNs are widely applied in computer vision and natural language processing [3]. Machine learning methods have made great progress in the field of fault diagnosis. For example, Gligorijevic et al. proposed a method for rolling bearing fault diagnosis. Through the five-level wavelet decomposition of the vibration signals, the standard deviations of the wavelet coefficients from six sub-bands were extracted as representative features; feature dimensionality reduction was then performed, and the diagnosis accuracy reached 98.9% [4]. However, some scholars gradually introduced CNNs into the field of fault diagnosis. By converting 1-D timeseries vibration signals into 2-D input matrices, some experts and scholars constructed 2-D convolutional neural network models for fault diagnosis of rotating machinery. Janssens et al. performed a short-time Fourier transform on the vibration information of rotating machinery, then input the transformed coefficient map into a constructed CNN model for feature extraction to achieve CNN-based multi-fault identification [5]. Jing et al. proposed an adaptive multi-sensor data fusion method based on deep convolutional neural networks, for fault diagnosis. The proposed method can learn features from raw data and adaptively optimize a combination of different fusion levels to effectively diagnose different faults of planetary gearboxes [6]. Wen et al. proposed a signal-to-image conversion method, using CNN techniques to extract features of the converted images, and achieved excellent test results for three famous datasets, comprising a motor bearing dataset, a self-priming centrifugal pump dataset, and an axial piston hydraulic pump dataset [7].

The main difference between the 1-DCNN and 2-DCNN lies in the dimension of input data and the sliding mode of the convolution kernel. Wu et al. proposed a 1-DCNN model for fault diagnosis of original vibration signals, for which test diagnosis accuracy reached 99.3% [8]. Huang et al. proposed a multi-scale cascade convolutional neural network (MC-CNN) model for fault diagnosis of bearings, achieving satisfactory results under non-stationary operating conditions [9]. He et al. combined a 1-DCNN and a LSTM (long short-term memory) network to construct a novel network model for fault diagnosis of bearings, with an average accuracy of over 99% [10].

For the traditional CNN, the convolution and pooling operations are carried out alternately, which reduces the feature maps’ size while increasing the receptive field. However, for the pixel-level prediction problem of image segmentation, the final feature output size is required to be consistent with the original image size, which involves the down-sampling and up-sampling processes, image resolution reduction, and information loss. To solve these problems, dilated convolution came into being [11]. Zhuang et al. proposed a stacked residual dilated convolutional neural network (SRDCNN) for real-time fault diagnosis of bearings by combining dilated convolution, LSTM, and residual networks, and their experimental results show that the proposed model has improved de-noising performance and adaptability [12]. Feng et al. used dilated convolution to replace conventional convolution and pooling structures, and introduced instance normalization (IN) to solve the issue of data style transfer. The proposed 1-D stacked dilated convolutional neural network (1D-SDCNN) model has an average accuracy of 96.8% for fault diagnosis of rolling bearings with variable loads [13]. Liang et al. combined LSTM, dilated convolution, and capsule networks to construct a new capsule network with gate-structure dilated convolutions (GDCCN). Their experimental results demonstrated that the proposed model has strong noise resistance and generalization in fault diagnosis of motors under variable load conditions [14]. As equipment has become more intelligent, complex, and integrated, it is difficult to accurately determine the characteristics of the failure status with a single feature. Moreover, using the 1-DCNN for fault diagnosis is highly dependent on fault datasets for roller bearing. The CNN fault diagnosis model can directly extract features from the original data to achieve end-to-end fault diagnosis without complicated data pre-processing. Dimensionality reduction methods, such as principal component analysis (PCA), cannot effectively preserve the time-dependence of timeseries data; moreover, information loss occurs in the process of dimensionality reduction.

In order to solve the above problems, this paper proposes a fault diagnosis method for the aircraft landing gear R/E system based on a 1-D dilated convolutional neural network (1-DDCNN). The main work of this paper is summarized as follows:

1. Analyze the aircraft landing gear R/E system’s main fault mode with its working principle.
2. A conventional 1-DCNN was constructed to classify faults based on the actuator’s displacement; experimental results show the average accuracy of the test set reached 91.80%.
3. The receptive field size affects the extraction of the original information by the model. There is a nonlinear relationship between the receptive field and the convolution kernel size for the expanded convolution. The convolution kernel size’s influence on classification accuracy is explored.
4. There is a low classification result with a single feature; consequently, 1-DDCNNselects multi-feature parameters with the system pressure, the pressure at the right and left end of the actuator cylinder. Experimental results reveal that the average test accuracy of the model reaches 99.80%.

## 2. The Typical Aircraft Landing Gear R/E System Analysis

### 2.1. System Working Principle and Composition

The landing gear system of a typical aircraft mainly includes the following: front landing gear and cabin door, main undercarriage and cabin door, landing gear R/E system, wheels and brakes, turning control system, landing gear position indication system, etc. Among them, the R/E system mainly completes normal R/E and emergency R/E functions, and provides the landing gear position indication signal.

**Figure 1:**
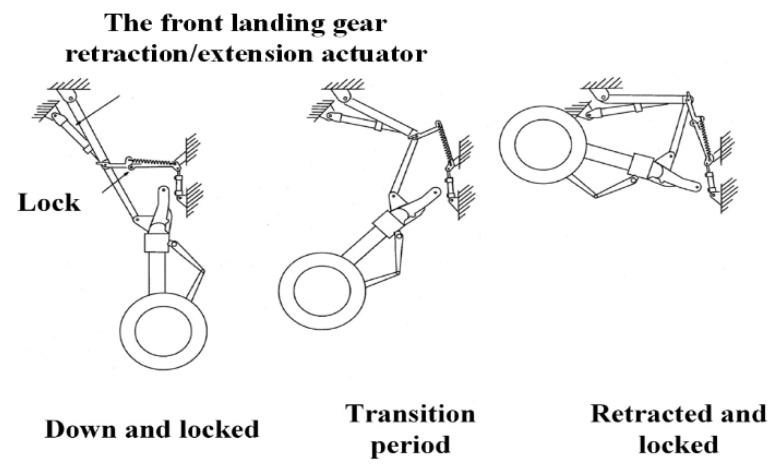
Front landing gear retracting process.

The front landing gear R/E process is similar to that of the main landing gear; therefore, only the front landing gear retraction process is described here. The working status of the front landing gear is shown in Figure 1. When the plane takes off, and the landing gear wheels are off the ground, the pilot sets the landing gear R/E control switch to the “UP” position, and the current flows to the landing gear R/E electromagnetic switch and the accumulator charging electromagnetic switch. The hydraulic fluid from the three-position four-way directional valve enters the front landing gear lower lock and actuating cylinder. As the accumulator charging solenoid switch is turned on, oil from the pump is supplied to the actuating cylinder, and the oil in the accumulator is also released to aid the landing gear retraction. When the aircraft is about to land, the pilot moves the control switch to the “DOWN” position, the three-position four-way directional valve is switched to the down circuit, and the fluid enters the front landing gear upper lock. Once the lock is opened, the oil enters the lowering chamber of the R/E actuator to lower the landing gear.

The landing gear R/E system mainly includes the following components: constant pressure variable pump, tank, hydraulic motor, filter, accumulator, actuator, press control, throttle valve, and three-position four-way directional control valve.

Through providing certain pressure and oil mass, the pump converts mechanical energy into hydraulic energy. The tank is used to store hydraulic oil. The filter is used to filter the hydraulic oil and remove its impurities. The accumulator not only supplies oil at both weak and heavy flows, it also compensates for leakage and maintains constant pressure. The actuator is a device that converts hydraulic energy into mechanical energy for linear reciprocating motion, which overcomes the load (including friction) and maintains the speed of motion using pressure-driven liquid flow. The relief valve is one of the common pressure valves used to regulate or limit the pressure in a hydraulic system. The throttle valve is a hydraulic component that regulates and controls the flow of oil in a hydraulic system. The function of the one-way throttle valve is to ensure that the oil flows in one direction, with no backflow, using the throttling effect. The solenoid directional valve is one of the frequently used hydraulic components in hydraulic systems, and is used to switch the direction of the hydraulic circuit. This article uses a three-position four-way solenoid directional valve. The “Position” refers to the working position of the spool. The “Way” marks the valve body of the oil port [15].

### 2.2. Failure Mode and Effect Analysis

The failure mode and effect analysis (FMEA), derived from typical civil aircraft design data in this subsection, can be used for parameter selection and fault injection into the simulation model, whereby fault datasets are obtained. The FMEA was carried out on the system’s main components for subsequent fault diagnosis, the results of which are shown in Table 1 below. The failure analysis in this paper focuses on the component level of the landing gear R/E system and does not explore the specific internal failure of each component. For the excessive noise from the pump, the failure threshold can be obtained by changing the air content of the oil. If the clogging of the throttle valves at both ends of the actuator cylinder has different effects on the system, it is necessary to change the throttle valve’s diameter at both ends of the actuator cylinder to obtain the failure threshold. Regarding the system failure caused by constant pressure variable pump leakage, a throttle valve should be connected to the constant pressure variable pump in parallel, and the throttle valve’s diameter should be changed to simulate different degrees of leakage of constant pressure variable pump. Actuator cylinder leakage also affects the normal operation of the system, and the failure threshold can be obtained by changing the actuator leakage coefficient.

## 3. 1-DDCNN Fault Diagnosis Model

### 3.1. 1-DCNN

**Figure 2:**
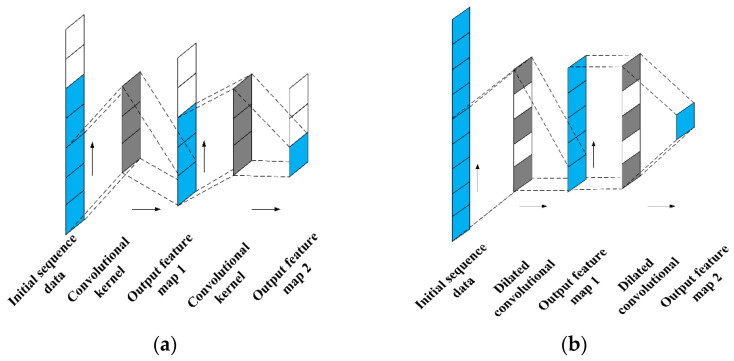
The receptive field of 1-DCNN. (**a**) 1-DCNN; (**b**) 1-DDCNN.

The receptive field is defined as the area size mapped on the original image by each pixel on the feature map output from each layer in the CNN. The neuron’s receptive field value decides the original range it can cover, meaning that it may contain more global features. Figure 2a shows the range of neuron receptive fields in the third layer of the 1-DCNN, with a convolutional kernel size of 3 × 1 and a step size of 1 × 1. The marked blue neurons in the third layer are mapped from the blue regions in the first layer, that is, the receptive field size of the input sequence data corresponding to a neuron in the output feature map 2 is 5 × 1.

Before inputting 1-D time series signals into 2-DCNN, the common method is to rearrange and combine signal sampling points, using a simple procedure, and convert them into 2-D matrix form. The 1-DCNN has the advantage that 1-Dtime series signals can be input directly without the need for cumbersome conversions.

The output receptive field of the n-th layer is:

(1) $$r_{n}=r_{n-1}+((k_{n}-1)\times \prod_{i=1}^{n-1}s_{i}),n\geq 2,$$

where $r_{n}$ is the receptive field size of the n-th layer,$k_{n}$ is the filter size of the n-th layer, and $s_{i}$ is the movement step size of the *i*-th layer filter.

According to the receptive field’s design principle, the size of the neuron receptive field in the last layer is close to the input signal’s length, that is, it satisfies the condition $r_{n}=L$, where L is the length of the input signal. The convolution kernel size is k, and the sliding step size of the convolution kernel is s. Each convolutional layer is followed by the maximum pooling layer, where the step size of the maximum pooling layer is $k_{pool}=2$, and the sliding window of the maximum pooling layer is $k_{pool}=2$. When n>2, the receptive field of the convolutional layer is:

(2) $$r_{n}=r_{n-1}+(k-1)\times \prod_{i=1}^{n-1}s_{i},$$

and the receptive field of the pooling layer is:

(3) $$r_{n+1}=r_{n}+(k_{pool}-1)\times \prod_{i=1}^{n-1}s_{i}\times s,$$

when n>2, the n-th network layer is a convolutional layer and n is odd, then the difference between the front and back receptive fields is $2^{n-1}$; thus, the expression for the receptive field $r_{n}$ of the neurons in the last pooling layer at the input signal, when *n* is even number, is:

(4) $$r_{n}=k+1+2k+4k+\cdots +2^{\frac{n}{2}-1}k=1+2^{\frac{n}{2}}k-k$$

According to the condition $r_{n}=L$, the value of k is obtained from:

(5) $$k\approx \frac{L-1}{2^{\frac{n}{2}}-1},$$

### 3.2. 1-DDCNN

Dilated convolution is also called expanded convolution; it replaces the traditional CNN pooling operation by introducing the expansion ratio, which can completely retain the feature information in the original signal so that the convolution kernel of the same size can obtain a larger receptive field.

(6) $$\{\begin{array}{cc} r_{1}=k & \\ r_{n}=r_{n-1}+(k_{n}-1)l_{n}, & n\geq 2 \end{array},$$

where $r_{n}$ is the receptive field size of the n-th layer network structure, $k_{n}$ is the convolutional kernel size of the n-th layer network structure, and $l_{n}$ is the expansion rate of the n-th layer network structure.

Figure 2b shows the receptive fields’ range in the third layer of the 1-DDCNN (output feature map 2) for the first layer(input sequence data) and the second layer (output feature map 1). The convolutional kernel size is 3 × 1 (*k* = 3). The step size is 1 × 1. The expansion rate is 2 ($l_{1}=l_{2}=2$). The receptive field size in the second layer corresponding to output feature 2 is 5 × 1, and the receptive field size in the first layer is 9 × 1.

### 3.3. 1-DDCNN Fault Diagnosis Model Framework

**Figure 3:**
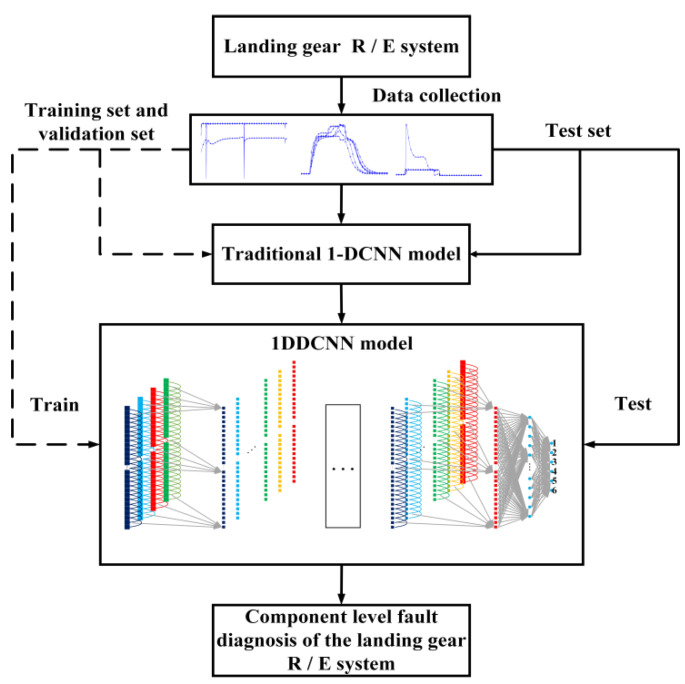
1-DDCNN fault diagnosis process.

The structural framework of the proposed fault diagnosis method based on the 1-DDCNN is shown in Figure 3. The model fault diagnosis process was as follows:

Step 1: Datasets were divided into training set, validation set, and test set.

Step 2: According to the structure and parameters of the traditional 1-DCNN model, the 1-DDCNN was preliminarily designed.

Step 3: The diagnostic accuracy of the multi-feature 1-DDCNN model under different convolutional kernel sizes was investigated to determine the final model hyper-parameters.

Step 4: The proposed model was trained and tested with a test set to obtain the fault diagnosis accuracy.

## 4. Experimental Implementations

### 4.1. Data Description and Operating Environment

**Figure 4:**
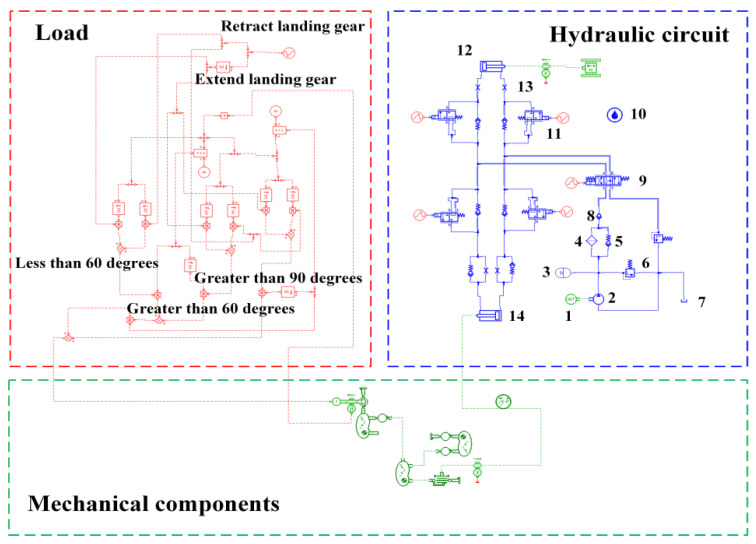
Simulation of landing gear R/E system. 1. Hydraulic motor; 2. Constant pressure variable pump; 3. Accumulator; 4. Filter; 5. Spring check valve; 6. Press control; 7. Oil tank; 8. Hydraulic check valve; 9. Three-position four-way directional control valve; 10. Hydraulic fluid; 11. Two-position three-way directional control valve; 12. Unlock actuator; 13. Flow control; 14. Actuatorcylinder.

Due to the fault data insufficiency regarding operation conditions, there is an incentiveto use AMESim^®^ to model the landing gear R/E system model and obtain fault datasets. According to the mutual logical relationship between components, the landing gear R/E system model was established, and is presentedin Figure 4. The blue section represents the hydraulic subsystem, the green section signifies the mechanical subsystem, and the red section denotes the external load of the system. Component parameter settings in the model are shown in Table 2.

The specific parameters of the FMEA in Section 2.2 are shown in Table 3.

**Figure 5:**
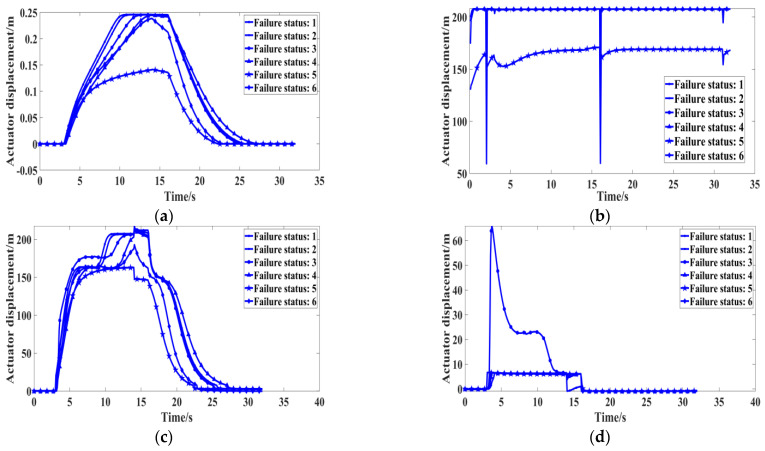
Comparison of the six fault types under different feature parameters. (**a**)The displacement of actuating cylinder; (**b**) system pressure; (**c**) the pressure at the right end of actuating cylinder; (**d**) the pressure at the left end of actuating cylinder.

The failure status:1 curve in the subgraphs a, b, c, and d of Figure 5 shows the main parameters’ variation trends under normal conditions, and that the entire landing gear R/E process time is 32 s, during which the landing gear retraction time is 7.5 s and the extension time is 10.8 s. These times are similar to those specified in the manual, and the manual requires that the R/E time shall not exceed the specified time by 1 s, or it will be regarded as a fault [16].

According to the fault thresholds in Table 3, 300 simulations were conducted for each of the six fault states, and the four parameters (actuator cylinder displacement, system pressure, and the pressure at the right and left end of the actuating cylinder) were sampled. The sampling frequency was set as 0.01 to obtain 1800 samples. Training, validation, and test sets were divided as 8:1:1, respectively. The details of the single-feature and multi-feature datasets are shown in Table 4 and Table 5, respectively, and the operation environment for the simulation is described in Table 6.

### 4.2. Experimental Model

Zhou [17] analyzed the following three important factors that have an impact on the performance of the CNN: network organization structure, network depth, and feature maps number. On the one hand, increasing the network depth can improve the recognition accuracy; on the other hand, increasing the feature maps number can also improve the recognition accuracy. Therefore, it is necessary to conduct a comparative study separately to determine the final model parameters. From Section 4.1, it is known that the sequence length of a single sample is 3201. The total number of convolutional and pooling layers is *n* = 12 (excluding the dropout layer). On the basis of Equation (5), the convolution kernel size of the first convolutional layer is 50. From the comparative test, the model with convolution kernel size 50, convolution number 4, and moving step size 1 at the first convolution layer, has the best diagnostic effect. The specific parameters of the traditional 1-DCNN model are shown in Table 7.

Due to the limited feature information extraction and inaccurate classification of the traditional 1-DCNN model with a single-feature parameter (actuator cylinder displacement), the three features, e.g., system pressure and the pressure at the right and left end of actuating cylinder, are selected to jointly characterize six failure statuses.

Referring to the traditional 1-DCNN, in 1-DDCNN we initially set the convolution kernel size as 50, the step size as 1, and the expansion factor as $l_{n}=2^{n-1}$, the calculation formula for the receptive field is:

(7) $$r_{n}=1+(k-1)(2^{n}-1)$$

**Figure 6:**
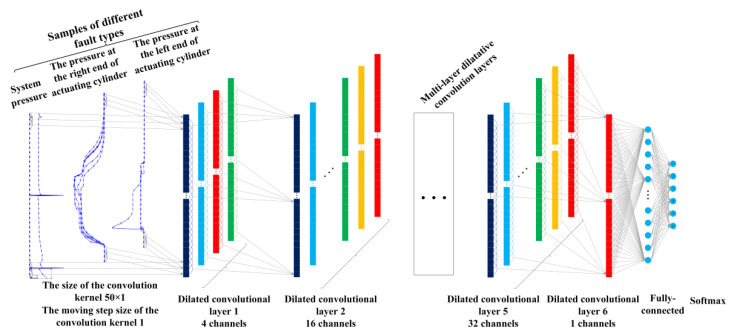
The structure of 1-DDCNN.

The network structure and initial settings are shown in Figure 6 and Table 8, respectively. The design principle of the model is that the output feature graph size of the last convolution layer is similar to, or exactly the same as, the size of the input data. The proposed 1-DDCNN has the following advantages: firstly, it constructs the convolutional kernel to obtain a larger receptive field and completely retain the feature information in the original signal; secondly, it can act as a dropout layer to prevent over-fitting.

### 4.3. Experimental Results and Analysis

#### 4.3.1. Research on the Size of Convolution Kernel of 1-DDCNN Model

According to Equation (7), once the expansion factor is determined, the parameter that has a decisive influence on the receptive field size is the convolution kernel size. Since the output size of each dilated convolution layer in the 1-DDCNN is 3201 × 1, the convolution kernel size does not affect the output features’ size of the dilated convolution layer, but has a great influence on the feature extraction degree of the original data. Therefore, it is necessary to investigate the convolutional kernel size’s effect on the classification accuracy. The convolution kernel size was set to 30, 40, 50, 60, and 70 in turn to investigate the diagnostic accuracy of the 1-DDCNN under different conditions, and to determine the final model hyper-parameters.

**Figure 7:**
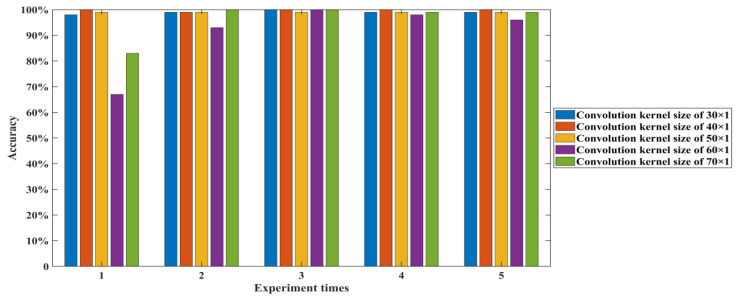
Test accuracy of 1-DDCNN under different sizes of convolution kernels.

Table 9 and Figure 7 show the detailed diagnosis results for the effect of convolution kernel size on the test samples in each trial. As the convolution kernel size increases, the total training parameters rise, and the model running time expands accordingly. The average accuracy of the 1-DDCNN with different convolution kernel sizes reached more than 90%. In particular, when the convolution kernel size was 40, the highest average accuracy of 99.80% was achieved in five training sessions. When the convolution kernel size was 50, its standard deviation was at least 0.0000, which indicates that the model had the highest stability under this condition.

**Figure 8:**
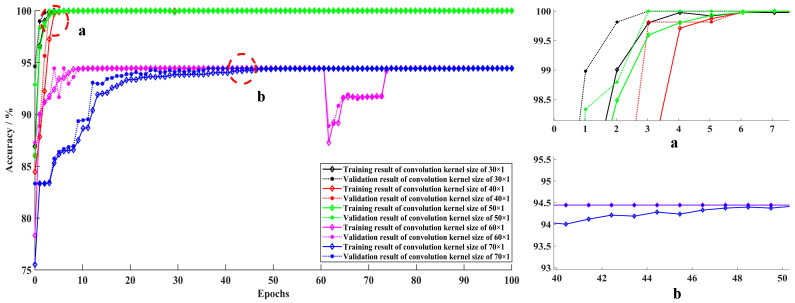
Training and validation accuracy at different convolution kernel sizes.

**Figure 9:**
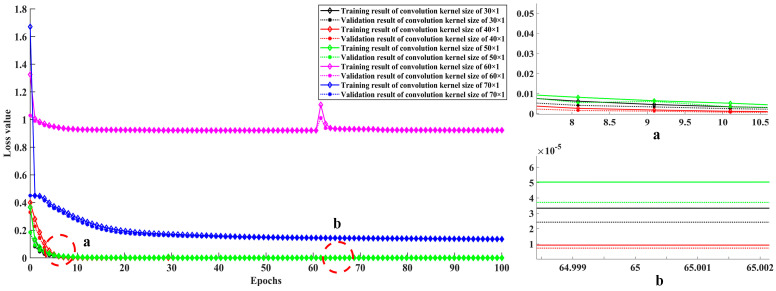
Training and validation loss values at different convolution kernel sizes.

Figure 8 shows that accuracies were close to 100% at the 10th iteration with convolution kernel size ranging from 30 × 1 to 50 × 1, and fluctuated around 94.5% from the 50th iteration onwards, with convolution kernel size ranging from 60 × 1 to 70 × 1. In fact, when the convolution kernel size is 60 × 1, the accuracy actually dropped. It can be seen from Figure 9 that the loss values within the convolution kernel size range of 30 × 1 to 50 × 1 approach 0 at the 10th iteration. The loss value at convolution kernel size 60 × 1 remained around 0.92 after 10th iteration, which indicates over-fitting. The loss value of the model corresponding to the convolution kernel size 70 × 1 remains around 0.14 after the 40th iteration. In particular, at the 65th iteration, the training and validation loss values at convolution kernel size 40 × 1 were both less than 1.0 × 10^−5^.

Considering the accuracy, stability, and training cost comprehensively, results are optimal when convolution kernel size is set as 40 × 1.

#### 4.3.2. Comparative Experiment of Three Models under Different Datasets

To show the dilated convolution’s advantages in feature extraction and information loss prevention, the comparative experiments of three models, e.g., traditional 1-DCNN, 1-DDCNN, and 1-DDCNN II, with dataset A and dataset B, were conducted. Depending on whether the same size is maintained between the feature map and the input data, two types of convolution operations exist: VALID (without padding) and SAME (with padding) convolution operations. Compared to the 1-DDCNN, the 1-DDCNN II’s dilated convolution layer was VALID, and the convolution kernel size was uniformly set to 51 × 1. Inputting the dataset into 1-DDCNN II, the output size of the flattening layer was 3264 × 1 after six dilated convolution layers, which is slightly larger than the sequence length of the input samples. The model structure is similar to the 1-DDCNN, and specific parameter settings are shown in Table 10.

**Figure 10:**
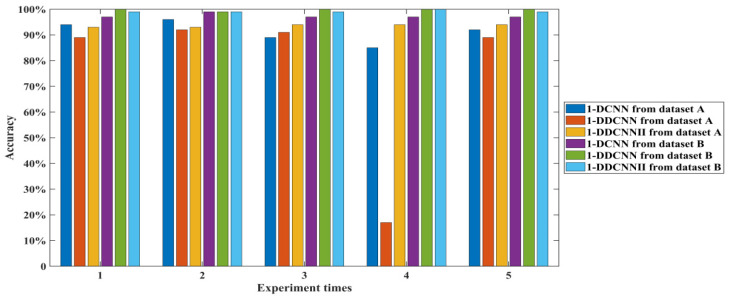
Test accuracy of different models from dataset A and dataset B.

It can be seen from Table 11 and Figure 10 that, compared with dataset A, the diagnostic accuracies of the 1-DCNN, 1-DDCNN, and 1-DDCNN II with dataset B were higher, and the total training parameters and training time increased slightly. The average accuracies of the 1-DDCNN and 1-DDCNN II reached more than 99%, and the standard deviation of both was 0.0045, which indicates that both models are stable.

**Figure 11:**
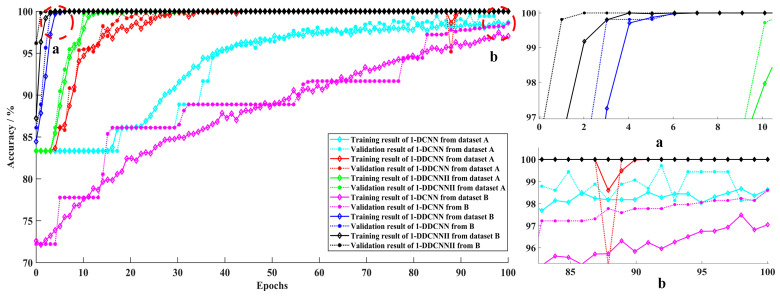
Training and validation accuracy of different models from dataset A and B.

**Figure 12:**
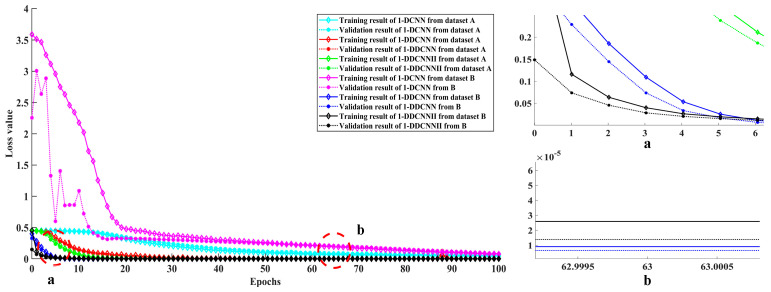
Training and validation loss value of different models from dataset A and B.

**Figure 13:**
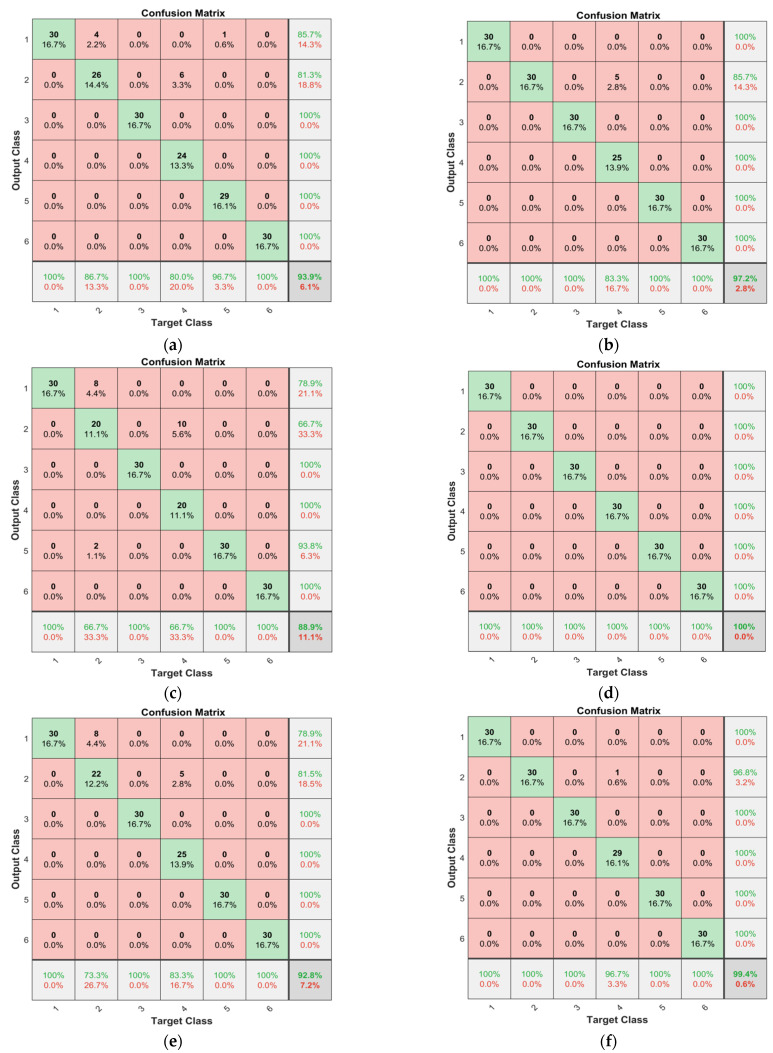
Confusion matrix of diagnosis results of different models based on dataset A and B.The following are the results of: (**a**) 1-DCNN from dataset A; (**b**) 1-DCNN from dataset B; (**c**) 1-DDCNN from dataset A; (**d**) 1-DDCNN from dataset B; (**e**) 1-DDCNNⅡfrom dataset A; (**f**) 1-DDCNNⅡfrom dataset B.

The training processes and confusion matrices for the three models are shown in Figure 11, Figure 12 and Figure 13.

In Figure 13, the rows represent the predicted class (output class) and the columns represent the true class (target class). The diagonal cells represent the observations that were correctly classified. The off-diagonal cells represent incorrectly classified observations. Both the observation number and the percentage of the total observation numbers, are shown in each cell. The far-right column in the plot shows the percentages of all the predicted examples belonging to each class that were correctly and incorrectly classified. These metrics are often called the precision and false rate, respectively. The bottom row in the plot shows the percentages of all the examples belonging to each class that are correctly and incorrectly classified. These metrics are often called the recall (or true positive rate) and false negative rate, respectively. The cell in the bottom right of the plot shows the overall accuracy.

When the 1-DCNN model with dataset A was located at the 100th iteration, the training accuracy was 98.63%, and the training loss value was 0.0441. The validation accuracy was 98.61% and the validation loss value was 0.0609. The confusion matrix corresponding to the test set is shown in Figure 13a; four fault samples caused by “excessive noise from the pump” were incorrectly classified as “normal”, and this corresponded to 2.2% of all 180 samples in dataset A. Similarly, six fault samples caused by “throttle valve blocking at left end of actuating cylinder” were incorrectly classified as “excessive noise from the pump”, and this corresponded to 3.3% of all data. Overall, 93.9% of the classifications were correct and 6.1% were wrong. It is believed that the potential reason for the identification error was the high similarities between sample sequences of different failure status, as shown in Figure 5a.

It can be seen in Figure 11 that the accuracies of both 1-DDCNNs with dataset B were close to 100% at the 10th iteration, while the accuracy of the traditional 1-DCNN model with dataset A and B did not reach 100%, even after 100 epochs, which indicates that the traditional 1-DCNN model loses a large amount of information during the pooling process.

Figure 12 shows the loss values of the 1-DDCNN and 1-DDCNN Ⅱ with dataset B are both close to 0 at the 10th iteration; the convergence trend of the loss value of the 1-DCNN with dataset B was slower than that of the other models. The training and validation loss value convergence curve of the 1-DCNN had a significant gap after the previous 20 iterations. In particular, at the 63th iteration, training and validation loss values of the 1-DDCNN with dataset B were both less than1.0 × 10^−5^. In terms of training cost, the iteration number should be set as 63 in subsequent model training.

It can be seen in Figure 13 that the test accuracies of subgraphs b, d, and f are higher than those of the subgraphs a, c, and e. In particular, the test accuracy of the 1-DDCNN (subgraph d) with dataset B reached 100%.

**Figure 14:**
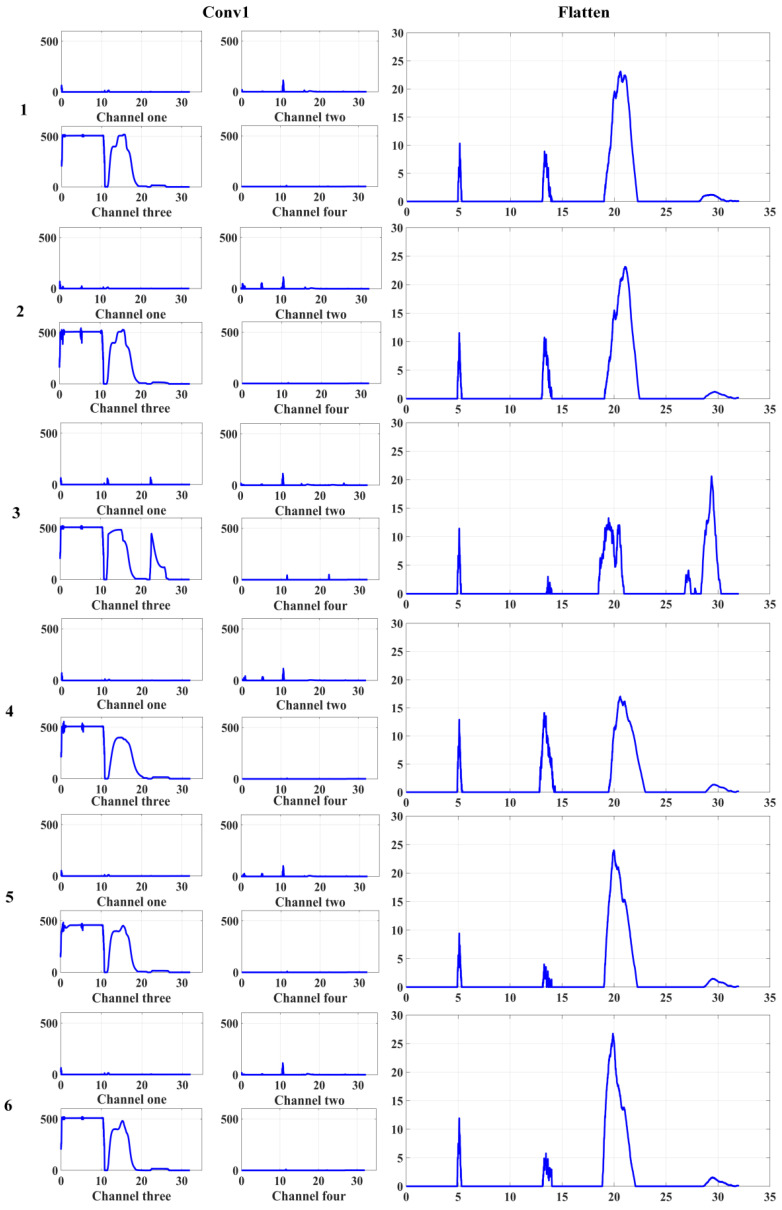
Visualization of output features of Conv1 and Flatten layers with the six failure statuses of 1-DDCNN. (**1**–**6**) correspond to the six failure statuses in Table 3.

Figure 14 shows the output features visualization of Conv1 and Flatten layers in the 1-DDCNN with six failure statuses. The feature similarity of the four-channel output of Conv1 was relatively high for the six failure statuses. After convolution and flattening operations, the six failure statuses exhibited unique characteristics, which are conducive to distinguishing the failure status of the model.

## 5. Conclusions

Since the 2-DCNN cannot directly process one-dimensional time series data, which often requires complex pre-processing, a novel 1-DDCNN is proposed for landing gear R/E system fault diagnosis in this paper. Dilated convolution can exponentially increase the receptive field of the convolution kernel by adding the convolution layer, which could acquire more redundant information to alleviate the influence of randomness. The displacement of the actuator cylinder was selected as the feature parameter, and the diagnosis classification was carried out on the traditional 1-DCNN model, for which the average diagnosis accuracy reached 91.80%. Due to the limited feature information extraction and inaccurate diagnosis for a single feature in the traditional 1-DCNN, multiple feature parameters are selected to jointly represent the fault and to input into the proposed model for feature integration. The convolution kernel size’s influence on classification accuracy is explored. When the convolution kernel size is 50, the model has the highe ststability. The results show that the average diagnostic accuracy of the proposed model is 99.80%, compared with other models.

Future work will be carried out on the following two aspects. Firstly, the system has noise in the actual working environment, and it is necessary to verify the robustness of the proposed model on noisy data. Secondly, this paper only considers the influence of a single parameter, such as oil mixing into the air or actuator leakage, on the system operating state. In the future, the complex situation of the simultaneous failure of multiple internal components, and the consequent effects on the system operating state, should be studied.

## Figures and Tables

**Table 1:** FMEA for the landing gear R/E system.

Components	Failure Mode	Failure Cause
Constant pressure variable pump	Insufficient oil output and pressure of the pump	Severe pump leakage or low pump speed
	Excessive noise from the pump	The pump is severely worn or air mixed in the oil
	Higher temperature	Abnormal wear in the pump
Actuatorcylinder	Piston rod stuck	The actuator cylinder is leaking seriouslyor press control is faulty
	The speed did not reach the standard value	The actuator cylinder is leaking seriously and it is subjected to excessive external load
	Creeping phenomenon	Air entered the actuatorcylinder
Directional control valve	Insufficient flow after reversing spool	Insufficient valve opening
	Spool stands still	Spool stuck or electromagnet failed
	Excessive pressure drops	Improper setting of some parameters
Filter	Filter clogging	Oil is contaminated
Throttle valve	The range of flow regulation is limited	Clogged throttle hole

**Table 2:** Component parameter settings in the landing gear R/E system model.

Sub-Model	Parameter	Value
Hydraulic fluid	Density	850 kg/m^3^
	Bulk modulus	1.7 × 104 bar
	Temperature	40 °C
	Air/gas content	0.1%
Constant pressure variable pump	Pump displacement	5 cc/rev
Hydraulic motor	Shaft speed	5000 rev/min
Filter	Equivalent orifice diameter	6 mm
	Critical flow number	2320
	Maximum flow coefficient	0.7
Accumulator	Polytrophic index	1.4
	Gas pre-charge pressure	118 bar
	Initial pressure	152 bar
	Accumulator volume	2 L
Actuatorcylinder	Piston diameter	69.596 mm
	Rod diameter	30.099 mm
	Length of stroke	276.1 mm
	Leakage coefficient	0 L/min/bar
Unlock actuator	Piston diameter	25.12 mm
	Rod diameter	11.049 mm
	Length of stroke	70.84 mm
Press control	Relief value cracking pressure	206.843 bar
	Relief value flow rate pressure gradient	20 L/min/bar
Throttle valve	Diameter	2 mm
Three-position four-way directional control valve	Valve natural frequency	80 Hz
	Valve damping ratio	0.8
	Flow rate	39 L/min
	Pressure drop	2.5 bar
	Valve rated current	40 mA

**Table 3:** Labels and failure threshold.

Failure Status	Labels	Failure Threshold
Normal	1	-
Excessive noise from the pump	2	Content of air in the hydraulic fluidis more than 5%
Throttle valve blocking at right end of actuating cylinder	3	The diameter of the throttle valve is less than 2.5 mm
Throttle valve blockingat left end of actuating cylinder	4	The diameter of the throttle valve is less than 2 mm
Constant pressure variable pump leakage	5	The diameter of the throttle valve is more than 3 mm
Actuator cylinder leakage	6	Leakage coefficient of actuator cylinder is greater than 0.01

**Table 4:** Single-feature dataset A.

Data Division	Failure Status					
	1	2	3	4	5	6
Training set	240	240	240	240	240	240
Validation set	30	30	30	30	30	30
Test set	30	30	30	30	30	30

**Table 5:** Multi-feature dataset B.

Data Division	Feature Parameters	Failure Status					
		1	2	3	4	5	6
Training set	System pressure	240	240	240	240	240	240
	The pressure at the right end of actuating cylinder	240	240	240	240	240	240
	The pressure at the left end of actuating cylinder	240	240	240	240	240	240
Validation set	System pressure	30	30	30	30	30	30
	The pressure at the right end of actuating cylinder	30	30	30	30	30	30
	The pressure at the left end of actuating cylinder	30	30	30	30	30	30
Test set	System pressure	30	30	30	30	30	30
	The pressure at the right end of actuating cylinder	30	30	30	30	30	30
	The pressure at the left end of actuating cylinder	30	30	30	30	30	30

**Table 6:** Experimental operation environment.

Operating Environment	Version
CPU	Intel(R) Core(TM) i5-9300H 2.40GHz
System	Windows 10 Home Chinese Version
Interpreter	Anaconda2.0.3
Compiler	PyCharm2019.3.4
Deep learning framework	Tensorflow2.0

**Table 7:** 1-DCNN model details.

No.	Layer Type	Kernel Size/Stride	Kernel Number	Output Size (Width × Depth)	Padding	Dropout Rate
1	Convolution1	50 × 1/1 × 1	4	3201 × 4	Yes	-
2	MaxPooling1	2 × 1/2 × 1	4	1600 × 4	No	-
3	Convolution2	3 × 1/1 × 1	16	1600 × 16	Yes	-
4	MaxPooling2	2 × 1/2 × 1	16	800 × 16	No	-
5	Dropout1	-	-	800 × 16	-	0.2
6	Convolution3	3 × 1/1 × 1	16	800 × 16	Yes	-
7	MaxPooling3	2 × 1/2 × 1	16	400 × 16	No	-
8	Convolution4	3 × 1/1 × 1	32	400 × 32	Yes	-
9	MaxPooling4	2 × 1/2 × 1	32	200 × 32	No	-
10	Dropout2	-	-	200 × 32	-	0.2
11	Convolution5	3 × 1/1 × 1	32	200 × 32	Yes	-
12	MaxPooling5	2 × 1/2 × 1	32	100 × 32	No	-
13	Dropout3	-	-	100 × 32	-	0.2
14	Convolution6	3 × 1/1 × 1	64	100 × 64	Yes	-
Activation function of the convolution layer: Relu						
Activation function of fully-connected layer:Softmax						
Optimization: Adam(1.0 × 10^−5^)						
Iteration: 100						

**Table 8:** 1-DDCNN model details.

No.	Layer Type	Kernel Size/Stride	Kernel Number	Expansion Factor	Output Size (Width × Depth)	Padding	Receptive Field
1	Convolution 1	50 × 1/1 × 1	4	1	3201 × 4	Yes	50
2	Convolution 2	50 × 1/1 × 1	16	2	3201 × 16	Yes	148
3	Convolution 3	50 × 1/1 × 1	16	4	3201 × 16	Yes	344
4	Convolution 4	50 × 1/1 × 1	32	8	3201 × 32	Yes	736
5	Convolution 5	50 × 1/1 × 1	32	16	3201 × 32	Yes	1520
6	Convolution 6	50 × 1/1 × 1	1	32	3201 × 1	Yes	3088
7	Fully-connected	256	1	-	256 × 1	-	-
8	Softmax	6	1	-	6	-	-

**Table 9:** Fault diagnosis results of 1-DDCNN under different sizes of convolution kernels.

The Convolution Kernel Size	Average Accuracy (%)	Standard Deviation	Total Parameter	Average Running Time/s
30	99.00	0.0071	**878355**	**187.2152**
40	**99.80**	0.0045	897355	211.7023
50	99.00	**0.0000**	916355	231.7082
60	90.80	0.1355	935355	262.0429
70	96.20	0.0740	953355	280.4578

**Table 10:** 1-DDCNN II details.

No.	Layer Type	Kernel Size/Stride	Kernel Number	Expansion Factor	Output Size (Width×Depth)	Padding	Receptive Field
1	Convolution 1	50 × 1/1 × 1	4	1	3151 × 4	No	51
2	Convolution 2	50 × 1/1 × 1	16	2	3051 × 16	No	151
3	Convolution 3	50 × 1/1 × 1	16	4	2851 × 16	No	351
4	Convolution 4	50 × 1/1 × 1	32	8	2451 × 32	No	751
5	Convolution 5	50 × 1/1 × 1	32	16	1651 × 32	No	1551
6	Convolution 6	50 × 1/1 × 1	64	32	51 × 64	No	3151
7	Fully-connected	256	1	-	256 × 1	-	-
8	Softmax	6	1	-	6	-	-

**Table 11:** Diagnosis results of different models.

Models	Datasets	Average Accuracy (%)	Standard Deviation	Total Parameter	Average Running Time/s
1-DCNN	A	91.80	0.0399	**833074**	**60.9052**
1-DDCNN		75.60	0.3278	897035	191.7420
1-DDCNNⅡ		93.60	0.0055	1036854	153.4254
1-DCNN	B	97.40	0.0089	833474	76.4105
1-DDCNN		**99.80**	**0.0045**	897355	211.7023
1-DDCNN Ⅱ		99.20	**0.0045**	1037262	171.7298
